# Insecticide Resistance Associated with* kdr* Mutations in* Aedes albopictus*: An Update on Worldwide Evidences

**DOI:** 10.1155/2018/3098575

**Published:** 2018-08-05

**Authors:** Michelangelo Auteri, Francesco La Russa, Valeria Blanda, Alessandra Torina

**Affiliations:** Laboratory of Entomology and Control of Environmental Vectors, Istituto Zooprofilattico Sperimentale della Sicilia, Via Gino Marinuzzi 3, 90129 Palermo, Italy

## Abstract

Insecticide resistance is an increasing problem worldwide that limits the efficacy of control methods against several pests of health interest. Among them,* Aedes albopictus* mosquitoes are efficient vectors of relevant pathogens causing animal and human diseases worldwide, including yellow fever, chikungunya, dengue, and Zika. Different mechanisms are associated in conferring resistance to chemical insecticides. One of the most widespread and analysed mechanisms is the knockdown resistance (*kdr*) causing resistance to DDT and pyrethroids. The mechanism is associated with mutations in the voltage sensitive sodium channel, which is involved in beginning and propagation of action potentials in nervous cells. The mechanism was originally discovered in the housefly and then it was found in a large number of arthropods. In 2011, a* kdr* associated mutation was evidenced for the first time in* A. albopictus* and afterward several evidences were reported in the different areas of the world, including China, USA, Brazil, India, and Mediterranean Countries. This review aims to update and summarize current evidences on* kdr* in* A. albopictus*, in order to stimulate further researches to analyse in depth* A. albopictus* resistance status across the world, especially in countries where the presence of this vector is still an emerging issue. Such information is currently needed given the well-known vector role of* A. albopictus* in the transmission of severe infectious diseases. Furthermore, the widespread use of chemical insecticides for control strategies against* A. albopictus *progressively lead to pressure selection inducing the rise of insecticide resistance-related mutations in the species. Such event is especially evident in some countries as China, often related to a history of uncontrolled use of chemical insecticides. Thus, a careful picture on the diffusion of* kdr* mutations worldwide represents a milestone for the implementation of control plans and the triggering of novel research on alternative strategies for mosquito-borne infections.

## 1. Introduction

### 1.1. *Aedes albopictus*: Vector Role, Ecology, and Control Strategies

*Aedes albopictus* (Skuse) (Diptera: Culicidae) is a diurnal mosquito native to the tropical and subtropical region of southern Asia [1]. It is among the most invasive species in the world, being reported across all continents except Antarctica [2]; the species is a well-recognized vector of a series of pathogens transmitting disease with evident public health importance, including yellow fever, chikungunya, dengue, and Zika [3, 4]. Since the original habitat of* A. albopictus* was a natural environment (i.e., edge of forest), it was classically described as a rural vector [5]. However, such species adapted to urban areas, breeding in artificial containers, where it progressively become a relevant vector of pathogens [6, 7].* A. albopictus* is in a rapid global expansion worldwide due to the reported resistance to desiccation of its eggs and to the increased trading in container shipment of tyres and plants [8, 9].

Regarding the major mosquito-borne viral diseases, chikungunya, dengue, and Zika are global relevant diseases across the world, from tropical regions to temperate ones [10]. Viruses associated with these diseases are transmitted primarily by* Aedes aegypti*, with different studies demonstrating the presence of viruses in field mosquito samples [10, 11]. However, the contributions of* A. albopictus* to such virus transmission are progressively increasing.

*A. albopictus* is involved in the transmission of dengue, which is the most prevalent disease worldwide with about 390 million cases reported per year [12]. Dengue virus was found in different field samples of* A. albopictus*, i.e., in Brazil [13, 14].

*A. albopictus* is also associated with the transmission of chikungunya virus, as demonstrated with its finding in field-collected specimens of* A. albopictus* sampled during the outbreak in 2007 in Italy (Ravenna), when more than 200 human cases occurred [15].

*A. albopictus* is also involved in the transmission of Zika virus with positive field samples reported in South America [16]. The first report of Zika virus was from a rhesus monkey in 1947 in Uganda [17]; the virus interferes with nervous system development, leading to microcephaly and Guillan-Barrè syndrome [18, 19]. In recent years, Zika virus in Brazil and French Polinesia caused various outbreaks [20, 21]. Given the increasing public health relevance of the* Aedes*-associated diseases, scientific efforts have been directed to the comprehension of the distribution and ecology of such vectors in the territories [22]. As previously mentioned, artificial and natural containers, as used tires, rock pools, and tree holes, were the most common habitats of* A. aegypti* and* A. albopictus* [23]. Temperature is a leading ecological factor influencing physiology of* Aedes* species, as gonotrophic cycle length and survival of adults [24, 25]. As an example, mortality increases as temperature exceeds 35°C [26]. However, studies on thermal limits revealed that* A. albopictus* was less tolerant to temperature changes than* A. aegypti* [27]. Furthermore, reproductive rate is closely associated with the relative humidity and vegetation canopy greenness, favouring protection from direct sunlight [28].

The adequate knowledge on* Aedes* ecology leads to the development of different control strategies, including classical approach as the control of immature mosquito stages or adults (insecticides), as well as innovative approaches as* Wolbachia*-based control and the Autodissemination Augmented by Males (ADAM) strategy. The latter ones based on the field-release of* Aedes* were contaminated with* Wolbachia* or pyriproxyfen, thus leading to a reduced transmission of viruses or reduced vector population, while other strategies include attractive toxic sugar baits, repellent use, traps for host-seeking, and gravid females [29].

However, the use of chemical insecticides for adult mosquitoes is still the most used method of control; various classes of insecticide have been extensively used, including dichlorodiphenyltrichloroethane (DDT), pyrethroids (i.e., deltamethrin, permethrin), carbamates, and organophosphates. In particular, pyrethroids are still the most used adulticides for* Aedes* mosquitoes [30].

However, the increased use of such compounds in worldwide areas where* Aedes* are recognized as public health threats leads to the progressive development of chemical insecticide resistance (IR) among mosquitoes [31]. IR represents a major risk for public health, potentially leading to failure in National Control Programs and uncontainable outbreaks [29, 30]. The problem was recently discussed during the first International Workshop on Insecticide Resistance in Vectors of Emerging Arboviruses: Challenge and Prospects for Vector Control, organized in 2016 by the Worldwide Insecticide Resistance Network [29]. Reports for* A. aegypti* and* A. albopictus* pyrethroids resistance status are progressively emerging, revealing a complex picture of mechanisms involved and local specific status. In particular, information on* A. albopictus* IR is still fragmented [30, 32]. Resistance to pyrethroids of* A. albopictus *was reported mainly in Asia and Americas, while less data are available for Africa and Europe [32]. However, in Mediterranean regions as Spain and Italy,* A. albopictus* resistant populations were detected [33, 34]. In Italy, a further relevant chikungunya outbreak of summer 2017 [35] again pointed out the attention on mosquito-borne diseases.

In such context, the aim of this review was to summarize current evidence on IR of* A. albopictus*, with a particular focus on knockdown resistance (*kdr*) conferring pyrethroids insensitivity. A systematic and updated knowledge represents a necessity to prompt novel researches aiming to depict a complete picture of* A. albopictus* IR status worldwide, especially in temperate regions where this vector may be still an emerging issue.

## 2. Insecticide Resistance

### 2.1. Mechanisms of Insecticide Resistance

The increasing use of chemical insecticide led to an expanding population of resistant mosquitoes. The Insecticide Resistance Action Committee (IRAC) defines resistance as “the selection of a heritable characteristic in an insect population resulting in the repeated failure of an insecticide product to provide the intended level of control when used as recommended” [36].

Four different categories has been defined including the different documented resistance mechanisms:

- *Metabolic resistance*, due to an increased detoxification caused by the overexpression or conformational changes of the enzymes involved in the chemical insecticide metabolism, sequestration, and excretion. P450-monooxygenases, glutathione S-transferases, and carboxy/cholinesterases are the main enzymes involved in this process [30, 37, 38].
- *Target-site resistance*, caused by a modification of the chemical insecticide site of action reducing or preventing insecticide binding at that site. Mutations in the voltage sensitive sodium channel (*Vssc*) gene are one of the most common causes of target-site resistance.
- *Reduced penetration*, due to modifications in the insect cuticle or digestive tract linings that limit the chemical insecticides absorption. However, the mechanism remains poorly understood, and its importance in* Aedes* species is yet to be confirmed [30].
- *Behavioural resistance*, which includes modifications in insect behaviour that help to avoid the lethal effects of chemical insecticides. This is considered a contributing factor that allows the insects to avoid the lethal dose of an insecticide.

### 2.2. Target-Site Resistance: The Knockdown Resistance (*kdr*)

One of the most important resistance mechanisms to pesticide is the knockdown resistance (*kdr*), first described in the house flies [39].* Kdr* has been documented globally in a large number of arthropods of relevant agricultural and health interest. In particular, DDT and pyrethroids trigger* kdr *phenotype [40]. DDT belongs to the organochlorine class and it exerts acute toxic effects through the hyperexcitation in the nervous system [37]. Pyrethroids are synthetic analogues of pyrethrine, used worldwide as broad-spectrum chemical insecticides with a neurotoxic activity [41]. Their extensive use is related not only to their efficacy but also to the limited effects on mammals [41]. Based on the absence or presence of a *α*-cyano group and on other characteristics, pyrethroids are differentiated in Type I or Type II [42, 43]. Other pyrethroids exhibit intermediate types of action [44]. Mostly used pyrethroids are permethrin, deltamethrin, cypermethrin, and cyfluthrin for residual and space spray treatments. Deltamethrin is also used in lethal ovitraps, reducing both adult and larvae densities [44].

The* kdr* is due to mutations in the voltage sensitive sodium channel (VSSC) or voltage-gated sodium channel (VGSC) encoded by the* Vssc* gene. The channel is involved in beginning and propagation of action potentials in excitable cells, as nervous cells. Major studies on VSSCs have been conducted in mammals, where the channel is formed by a main *α*-subunit of 260kDa and smaller *β*-subunits of about 30-40 kDa [45]. *α*-Subunit contains four homologous domains (I-IV) with each domain characterized by six transmembrane segments (S1-S6), as described by Loughney and colleagues [46]. In mammals, at least nine sodium channel genes are present [47], while in insects only one sodium channel gene has been found [48].

**Figure 1:**
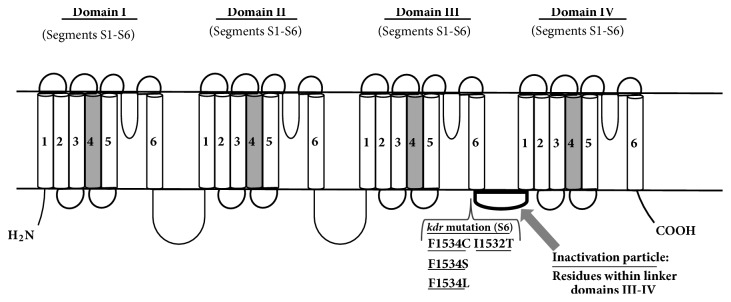
*Schematic representation of the voltage-gated sodium channel (VGSC) with major kdr mutations discussed in the present work*. S1–S4 segments: voltage-sensing domains; S5-S6 segments and connecting loop: pore-forming domains. Physiologic mechanism: (i) after depolarization, S4 segments (in grey) open the gate moving outward (activation). (ii) Then, residues in the linker domains III-IV constitute an inactivation particle allowing channel inactivation (inner pore block). (iii) Following repolarization, the S4 segments close the gate (deactivation).

The S1–S4 segments act as voltage-sensing domain, while the S5 and S6 segments and the loop connecting them constitute the pore-forming domains. A small intracellular linker between the S4 and S5 segments connects the voltage-sensing domain to the pore-forming domain [49] (Figure 1).

Sodium channels activates following opening and then undergoes inactivation and deactivation (closing). In response to membrane depolarization, the positively charged S4 segments move outward opening the gate (active state). Opening is followed by the inactivation of the channel due to the block of its inner pore by an inactivation particle (residues in the linker of domains III-IV). Subsequent to repolarization, the S4 voltage sensors move backward and close the activation gate, leading to the deactivated state of the channel [49].

DDT and pyrethroids alter the gating kinetics of VSSC binding the activated form of the channel and blocking it in the open status. Thus, they disrupt the physiological transmission of the nervous impulse [50]. Binding sites for chemical insecticides in the channel are not well defined at the molecular level and mechanisms through which they block the sodium channel in the open state are not well known [51, 52]. However, recent studies started to identify possible chemical insecticide binding sites [53, 54]. Using the* A. aegypti* AaNav1-1 sodium channel as model, the necessity of a simultaneous binding to two receptor sites has been revealed to lock the channel in the open state, thus disrupting nervous system signalling [53]. Another study on an open-state model of insect sodium channel has showed binding of aromatic moieties of insecticides to 3 sites including a sodium ion in central cavity, a portion delimited by IIS6, IVS6 IIIP1, and the pore helix IP1 [54]. Such studies underline the actual complexity of insect sodium channels and scientific efforts should be directed to an effective comprehension of their physiology in order to understand insecticide actions and efficacy.

### 2.3. Main Mutations in the* Vssc* Gene

Several different* Vssc* mutations have been reported and many of them have been found even in* Aedes* mosquitoes [32]. Most of them are located in the transmembrane segments IIS5, IIS6, and IIIS6. Even linker regions connecting S4-S5 of domain II have been associated with substitutions [32, 49].* Vssc* mutations reduce the sensitivity to DDT and pyrethroids, especially of type I pyrethroids. Many of them are close to the pyrethroid binding site of the receptor, while others are not situated in this functional site and their mechanism of inactivation is unknown. Characterizations of the mutations conferring pyrethroid resistance have implications in both basic and applied aspects of research, contributing also to the better understanding at a molecular level of the action mechanism of the sodium channel [49, 55, 56]. Historically, the first mutation related to the* kdr* was found in* Musca domestica* at the position 1014 of IIS6; the mutation was a substitution of the leucine with a phenylalanine, L1014F [57]. This mutation was subsequently frequently found in other several insects, as well as mosquitoes, in particular in* Anopheles gambiae* [58]. Moreover, this site was found to be affected by a series of divergent substitutions leading to the change of this leucine 1014 with F, C, H, S, or W [59–61]. However, not all the substitutions reported in each of the above-mentioned sites have been related to a channel loss of sensitivity versus pyrethroids.

In mosquitoes, especially in* Aedes aegypti*, several mutations have been identified that can be present or individually or simultaneously in the same genome, with the position numeration following the amino acid sequence of the most abundant splice variant of* Musca domestica* sodium channel [62].  Table 1 summarizes the current knowledge on the principal mutations occurring in the sodium channel of* A. aegypti* [44, 62]. Different substitutions occurring simultaneously in the same genome significantly reduce sensitivity to pyrethroids. As an example, the combination of S989P and V1016G mutations greatly increases VSSC insensitivity to pyrethroids. The additional presence of F1534C mutation further enhances the insensitivity to both deltamethrin and permethrin [44].

Pyrethroid resistance in* Aedes* species is a global problem. Resistance in* A. aegypti* has been more thoroughly studied and widely reviewed [32]. However, since the first report occurred in 2011 [62], an increasing number of cases of pyrethroid resistance have been identified even in* A. albopictus* populations.

## 3. Evidences of* kdr* Mutations in* A. albopictus* Worldwide

### 3.1. First Reports of F1534C and F1534L* kdr* Mutations in* A. albopictus*

The first* kdr* mutation in* A. albopictus* was found in a mosquito population collected in 2009 in Singapore [62]. Authors genotyped 5 loci within the VGSC (S989P, I1011M or V, V1016G or I, F1534C, and D1763Y) and they identified a mutation at codon 1534 (F1534C mutation: TTC to TGC, phenyalanine to cysteine), in domain III, segment 6. They found the F1534C mutation in 24 out of 26* A. albopictus* mosquitoes, with 14/26 specimens homozygous for the mutation. F1534C was specifically associated with Type I pyrethroid-permethrin resistance in* A. aegypti* [63]. Thus, the analysed mosquito population might phenotypically display a permethrin resistance although no susceptibility bioassays were performed on such population. In Singapore region, the fixation of F1534C mutation could derive from widespread permethrin-based treatments for pest control within dengue prevention programs.

The second* kdr* mutation in* A. albopictus* was revealed* via* a survey in United States in 2011 [64]; populations of mosquitoes from New Jersey and Florida showed a resistant phenotype to DDT in WHO tube test bioassays. High *α*- and *β*-ESTs and GSTs activities were found in Florida populations, remarking the role of metabolic mechanism in chemical insecticide resistance in such populations. A Florida specimen also showed a novel* kdr* mutation in codon 1534 (F1534L mutation: TTC to TTG, phenyalanine to  leucine). The study revealed a selection of DDT-resistant mosquitoes in USA in absence of a massive DDT-based pest control (DDT utilization was terminated in 1972 in USA). Thus, a recent introduction of population of DDT-resistant* A. albopictus* from Asia may be supposed, as also suggested by studies on* A. albopictus* chemical insecticide resistance in Africa [65]. Novel researches will allow pointing out the relations between possible chemical insecticide resistance drivers in United States.

### 3.2. A Multicountry Survey for* kdr* Mutations in* A. albopictus*: First Depiction of Global Evidences and Identification of Two Novel* kdr* Mutations

The discovery of* kdr* mutations in* A. albopictus *rapidly prompts the research efforts for the definition of its impact all over the possible target countries where pest control programs were implemented. A multicountry survey [66] was conducted analysing population of* A. albopictus *caught from 2011 to 2014 in 12 sites from 6 countries (Japan, China, Singapore, USA, France, and Italy). VGSC gene sequences of domains II, III, and IV were analysed, and nonsynonymous mutations were revealed at codons 1532 and 1534 (domain III). In particular, populations of* A. albopictus* from Italy displayed a novel I1532T mutation and a F1534L mutation [66]. Regarding codon 1534, the reported mutations varied among the countries sampled: in Southern China, in addition to the F1534L mutation, a novel F1534S mutation was reported. F1534S mutation was also detected in mosquitoes from Florida (USA). The survey also revealed the presence of F1534C mutation in Greece. In addition to the nonsynonymous mutations within domain III, a total of 29 synonymous mutations were found across domains II, III, and IV.

The novel I1532T mutation was not detected in other Italian sites, underlining the importance of site-specific factors (i.e., climate, disposable breeding sites, methods, and frequency of pest control) in the development of resistant mosquito populations. Distribution of nonsynonymous mutations revealed a patchy pattern, including sites with absence of such mutations along with sites exhibiting polymorphism at codon 1532 or 1534 (China, Italy, Greece, and Florida). Such irregular pattern points out the complex mechanisms and factors affecting pyrethroid resistance in* A. albopictus. *A correct analysis of* kdr* mutations within an area should include a large number of mosquito samples from different environments and a comprehensive report on intervention campaigns for pest control. This could allow correlation of experimental observations with the peculiar features of the territories.

In addition to genotypization, Xu et al. [66] perform phenotypization assays for insecticide susceptibility on the Southern China populations of* A. albopictus via* the standard WHO protocol [67], obtaining a significant increase in knockdown time in field mosquitoes populations compared to control (laboratory) populations. Following this, genotypization of 1534 codon from the identified phenotypically resistant mosquitoes revealed a significant association between the novel F1534S mutation and deltamethrin resistance. The association suggests that* kdr* mutation may represent a useful biomarker for* A. albopictus* resistance to pyrethroids.

### 3.3. Further Surveys on* kdr* Mutations

Besides the aforementioned multicountry survey, different studies on* kdr* mutations in* A. albopictus* were conducted from Asia to Africa and America.

#### 3.3.1. Malaysia, Costa Rica, and Central African Republic (CAR)

In Malaysia [68], phenotypization studies on* A. albopictus* samples collected in 2010 showed a high susceptibility to pyrethroids (except for a population in Kuala Lumpur). Instead, collected* A. aegypti* specimens were highly resistant to permethrin, deltamethrin, and DDT. In* A. aegypti, *resistance may derive from both* kdr* mutations (F1534C mutation) and P450-related metabolic resistance. In the resistant* A. albopictus* population, metabolic pathways could play a major role since no* kdr* mutations were detected. Such difference could be related to a confinement of* A. albopictus *in rural areas in Malaysia compared to the urban setting of* A. aegypti*. These conditions lead to different exposure to pyrethroids insecticides and selection pressure.

In Costa Rica (Central America) [69],* A. albopictus* is a recent invasive species. A study in specimens collected in 2014 [69] reported only a silent mutation at loci V1016, while neither the common F1534C* kdr* mutation nor the F1534L mutation was found. Such results are in line with the sampling site (an organic farm free of chemical insecticide use) and the fitness cost of insecticide resistance for mosquitoes [70], making unlikely the development of insecticide resistance in a new-invading species.

In CAR [71], a survey conducted in 2014 showed a resistant phenotype in* A. albopictus* populations towards DDT, while susceptibility to carbamate or organophosphate. Specimens displayed an increased activity of enzyme systems as *α*- and *β*-esterase, with no* kdr *mutations. Considering the recent introduction of* A. albopictus* in CAR (2009), the invasion by a population already resistant to pyrethroids could be assumed. A constant monitoring of such population should allow determining if metabolic resistance is the sole mechanism in CAR, as well as detecting possible* kdr* mutations. Moreover, CAR may represent a useful model to study the selection pressure for* A. albopictus* in presence of low levels of insecticide, given the lack of extensive chemical insecticide campaigns against* A. aegypti* and* A. albopictus* in the country.

#### 3.3.2. Brazil

In Brazil, an extended survey within dengue vector control programs was implemented from 2009 to 2014 [72]. The study detected the F1534C mutation for the first time in Brazilian* A. albopictus* populations, with an allele frequency from 0 to 10% in the Paranà State and 3% in Porto Velho. Municipalities displaying such mutation are important wide urban centers with a history of dengue outbreaks, and pyrethroid resistance of* A. aegypti* was already reported [73]. Thus, selection pressure due to chemical insecticide may be acting also on* A. albopictus*. However, since the mutated allele appeared in heterozygosis, mutation may be recently emerged, implicating an ongoing process of resistance development in* A. albopictus*.

#### 3.3.3. India

In India, the picture of* kdr* mutations in* A. albopictus* displays a unique complexity. In the country in 2016 about 129,000 dengue cases and 245 deaths were reported [74], making the clarification of the chemical insecticide resistant status of the* Aedes* vectors necessary for public health.

A study conducted in Indian urban areas in 2012 [75] reported a highly DDT-resistant phenotype in two* A. albopictus* mosquito populations. However, no insecticide resistance-related mutations in the* Vssc* gene were detected. Moreover, no analysis of enzymes possibly involved in metabolic resistance to chemical insecticides has been conducted, thus preventing from identifying possible causes of the resistance status of* A. albopictus* in Indian territories.

A more complete study conducted in India in 2015 [76]* via* WHO tube test assay reported different* A. albopictus* phenotypically resistant to DDT and suspected resistances to cyfluthrin. Distribution of wild-type and mutant (F1534C mutation) genotypes after DDT or cyfluthrin exposure identified wild-type (F/F1534), heterozygous (F/C1534), and homozygous mutant samples (C/C1534). Mutant allele frequency in survivor mosquitoes was 0.64 after DDT and 0.89 after cyfluthrin exposure, with a significant correlation between* kdr* mutations and DDT/cyfluthrin resistant phenotypes. In particular, survivor mosquitoes were not only mutant homozygous but also F/C1534 heterozygous. This observation suggests the contribution of additional mechanisms as metabolic resistance. In fact, biochemical assays in adults revealed higher activity for GST, esterases, and cytochrome P450 oxidase in resistant strains. Authors investigated only the F1534C* kdr* mutation; a broader survey on* kdr* mutations in* A. albopictus* may reveal the presence of further mutations and their association with chemical insecticide resistance.

A recent report [74] investigated the resistant status of* A. albopictus* in West Bengal, an Indian area with about 22,000 dengue cases reported in 2016. Phenotypization assay* via* the WHO test tube revealed DDT and deltamethrin resistant adult mosquitoes. Specimens exposed to deltamethrin were analysed for mutations in six loci of* Vssc* gene. None of the previously reported mutations [62] was found, while 11 synonymous mutations and a nonsynonymous mutation were present. However, such mutation was not associated with pyrethroid resistance since the specimens carrying it were susceptible to deltamethrin. Regarding DDT resistance in* A. albopictus*, the authors did not perform any study; thus, causes of DDT resistance in the sampled* A. albopictus* remain not understood.

Summarizing, a target-site resistance to DDT/pyrethroids is potentially emerging in* A. albopictus *in India, although the F1534C* kdr* mutation is rarely present. Different kinds of chemical insecticides are used within the National Vector Borne Disease Control Programme (NVBDCP) in India [74]; hence,* Aedes* species may be under selection pressure. Resistance of* A. albopictus* to DDT may represent the major issue for India, although other investigations on pyrethroid resistance and the analysis of detoxifying enzyme activity would be needed.

#### 3.3.4. China

In parallel with the participation in the aforementioned multicountry survey [66], a further investigation of* A. albopictus* resistance status in China was performed in the Guangdong region [77]. Here, 90% of dengue cases in China occurred and* A. albopictus* is the unique vector. The fast urbanization of the region along with extensive insecticide use leads to development of insecticide resistance in such species. Moreover, a relevant risk for Zika outbreak in Guangdong is present due to recently imported cases [78]. The study of Li et al. (2018) [77] focused on* A. albopictus* reared from collected larvae in six areas with different ecological features (urban, suburban, and rural). Such study organization allows a comparison of resistance status in areas differing for type and frequency of chemical insecticide use. Phenotypization bioassays showed that one rural and two urban mosquito populations were DDT resistant, while only one urban population was resistant to deltamethrin and the carbamate propoxur.* Kdr* genotyping of specimens after deltamethrin resistance bioassay or after DDT resistance bioassay detected nonsynonymous mutations in codon 1534 (domain III). In particular, F1534S and F1534L mutations were significantly associated with deltamethrin resistance; a trend towards a significant association between these mutations and DDT resistance was present, especially in urban populations. Enzyme activities assays showed in suburban population higher levels of P450 and GST. These enzymes may contribute to DDT/pyrethroid resistance in mosquitoes [36, 58, 59], along with* kdr* mutations. The study, comprehending collection of samples from ecologically different areas, the research of different* kdr* mutations, resistance bioassay, and evaluation of detoxifying enzyme activities, is a useful model for characterization of* A. albopictus* chemical insecticide resistance in a defined area.

A subsequent survey conducted on 2015 in Haikou City (Hainan Island), an area nearby the Guangdong region, detected the presence of two mutant 1534 codons in adult* A. albopictus* [79], which displayed a DDT resistance phenotype in the WHO tube bioassay. In addition,* A. albopictus* larvae showed resistance to pyrethroids (deltamethrin, permethrin, and beta-cypermethrin). Mutations F1534C, F1534S, and F1534L were reported with a significant correlation with deltamethrin or DDT-resistant phenotypes. In line with the previous findings [77], chemical insecticide susceptibility bioassay on* A. albopictus* larvae showed a resistant status of urban populations compared to the rural ones, correlated with a more intensive insecticide use in urban areas.

## 4. Conclusions

The increasing reports on mosquito chemical insecticide resistance worldwide lead to wide scientific efforts to identify major resistance mechanisms as well as to face up a potential public health emergency. Knockdown resistance represents a major issue which is spreading among different insects and particularly in mosquitoes vector of severe pathogens causing malaria, chikungunya, dengue, and Zika. A major focus on* Aedes aegypti* in recent years leads to an underestimation of the presence of* kdr* mutations in the other relevant vector* Aedes albopictus*. First evidences on* kdr* mutations in* A. albopictus* are accumulating across the worlds, especially in Asia and Americas but also in European territories, depicting a complex scenario for which further investigations are certainly needed.

Different from* Aedes aegypti*, where several different mutations have been found in domains II, III, and IV of the VSSC, mutations detected in* A. albopictus* are all associated with domain III. With regard to the involved segments, the S5 and S6 and the linker S5-S6 have been implicated in* A. aegypti*, while the sole S6 was reported as site of mutation in* A. albopictus*. This evidence suggests that the resistance would be related to mutations in amino acidic residues forming the pore structure of the channel and that they could probably diminish the receptor affinity for the chemical insecticide. In addition, it is worth noting that since the exact binding site for insecticides is not known and that current studies focused on analysis of few loci within the extensive sodium channel gene, the possibility of other mutations that could play a role in* A. albopictus* resistance to chemical insecticide should not be excluded.

**Figure 2:**
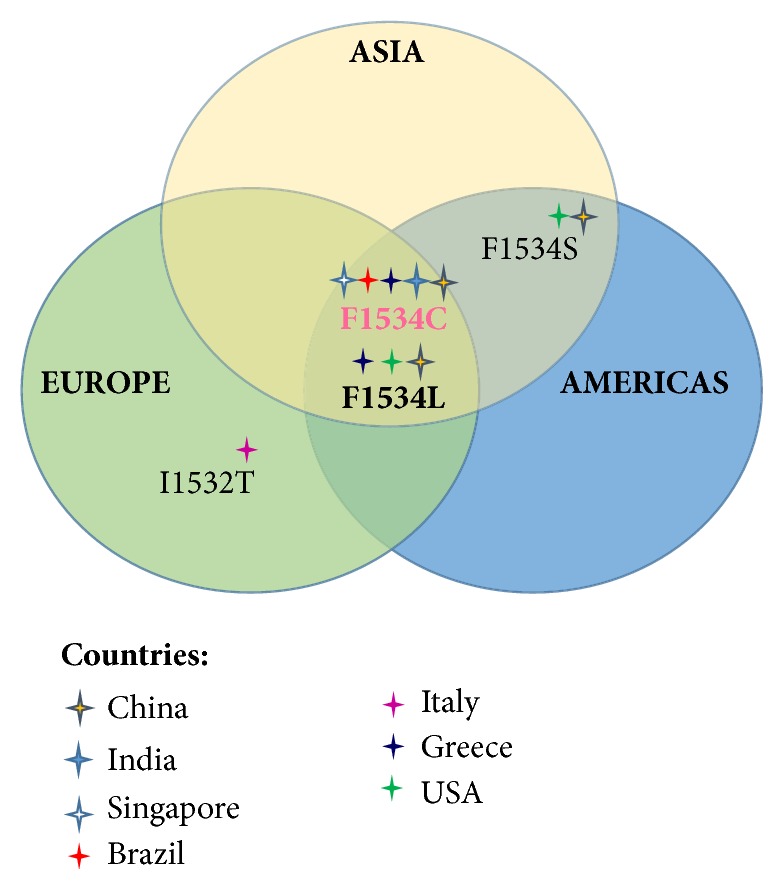
*Schematic representation of the geographical distribution of the kdr associated mutations identified worldwide in A. albopictus*. Symbol colour is related to the countries where the mutations have been found.

Since these mutations arise spontaneously and they are then selected in resistant populations, it is worth noting that in all the reported studies the mutations occurred almost exclusively in the same amino acid (Figures 1 and 2). This could be due to the following: (1) a particular susceptibility to mutations of this site; (2) the higher number of studies on* kdr* mutations conducted on* A. aegypti* compared to* A. albopictus. *This is related to the lower vectorial competence of* A. albopictus* compared to* A. aegypti*, and then to its consideration as minor vector; (3) the lower selective pressure to which* A. albopictus* was subjected compared to* A. aegypti*. Indeed,* A. albopictus* is a mosquito species that has undergone an urbanization process more recently. These considerations underline the need for further studies focused on this mutation site to better clarify pyrethroid insecticide mechanism of action. The increased knowledge may be followed by the discovery of new chemical insecticides effective for mosquitoes.

The realization of research networks and the planning of complete studies including samples collection from various ecological areas and correlation between WHO insecticide bioassays and* kdr* mutations analysis represent milestones for studies addressing chemical insecticide resistance.

## Figures and Tables

**Table 1:** Overview of principal regions of *A. aegypti* sodium channel where *kdr* mutations have been detected [44, 62].

**Mutation**	**Interested domain**	**Region**
S989P	domain II	linker connecting S5-S6
V1016G	domain II	S6
V1016I	domain II	S6
F1534C	domain III	S6
D1763Y	domain IV	S5
I1011V	Domain II	S6
I1011M	Domain II	S6
L982W	Domain II	Linker connecting S5-S6
T1520I	Domain III	S6
G923V	Domain II	S5
